# A genetic map of *Xenopus tropicalis*

**DOI:** 10.1016/j.ydbio.2011.03.022

**Published:** 2011-06-01

**Authors:** Dan E. Wells, Laura Gutierrez, Zhenkang Xu, Vladimir Krylov, Jaroslav Macha, Kerstin P. Blankenburg, Matthew Hitchens, Larry J. Bellot, Mary Spivey, Derek L. Stemple, Andria Kowis, Yuan Ye, Shiran Pasternak, Jenetta Owen, Thu Tran, Renata Slavikova, Lucie Tumova, Tereza Tlapakova, Eva Seifertova, Steven E. Scherer, Amy K. Sater

**Affiliations:** a Department of Biology and Biochemistry, University of Houston, Houston TX USA; b Department of Cell Biology, Charles University in Prague, Faculty of Science, Czech Republic; c Human Genome Sequencing Center, Baylor College of Medicine, One Baylor Plaza, Houston, TX USA; d Sanger Institute, Wellcome Trust Genome Campus, Hinxton, Cambridge CB10 1HH, UK

**Keywords:** *Xenopus*, *X. tropicalis*, Genetic map, Genome, Simple sequence length polymorphism

## Abstract

We present a genetic map for *Xenopus tropicalis*, consisting of 2886 Simple Sequence Length Polymorphism (SSLP) markers. Using a bioinformatics-based strategy, we identified unique SSLPs within the *X. tropicalis* genome. Scaffolds from *X. tropicalis* genome assembly 2.0 (JGI) were scanned for Simple Sequence Repeats (SSRs); unique SSRs were then tested for amplification and polymorphisms using DNA from inbred Nigerian and Ivory Coast individuals. Thus identified, the SSLPs were genotyped against a mapping cross panel of DNA samples from 190 F2 individuals. Nearly 4000 SSLPs were genotyped, yielding a 2886-marker genetic map consisting of 10 major linkage groups between 73 and 132 cM in length, and 4 smaller linkage groups between 7 and 40 cM. The total effective size of the map is 1658 cM, and the average intermarker distance for each linkage group ranged from 0.27 to 0.75 cM. Fluorescence *In Situ* Hybridization (FISH) was carried out using probes for genes located on mapped scaffolds to assign linkage groups to chromosomes. Comparisons of this map with the *X. tropicalis* genome Assembly 4.1 (JGI) indicate that the map provides representation of a minimum of 66% of the *X. tropicalis* genome, incorporating 758 of the approximately 1300 scaffolds over 100,000 bp. The genetic map and SSLP marker database constitute an essential resource for genetic and genomic analyses in *X. tropicalis*.

## Research highlights

► A genetic map of 2886 Simple Sequence Length Polymorphisms for *Xenopus tropicalis*. ► 10 major linkage groups corresponding to the 10 chromosomes, plus 4 minor linkage groups. ► Linkage groups are cytogenetically mapped to chromosomes by Fluorescence *In Situ* Hybridization.

## Introduction

*X. tropicalis* has emerged as a promising model system for genetic analyses of vertebrate development, extending the molecular, biochemical, and embryological strengths known from *Xenopus laevis* with the addition of genetic tractability. Initial genetic screens have yielded unique and interesting phenotypes (Abu-Daya et al., 2009; Goda et al., 2006, Grammer et al., 2005; Noramly et al., 2005), and the *X. tropicalis* genome assembly is now available (Hellsten et al., 2010). The integration of genetic and genomic approaches requires a genetic map for use in positional cloning of genes identified by mutation.

Although several types of polymorphic markers can be used to establish a genetic map, we chose Simple Sequence Length Polymorphisms (SSLPs), identified from an initial set of Simple Sequence Repeats (SSRs). Short, tandemly repeating sequences, SSRs are present at an average frequency of 1/20–40 kb (Strachan and Read, 2003); strain-specific differences in the length of the repeat are reflected as differences in the size of a PCR fragment that encompasses the SSR.

SSLPs offer several advantages. Unlike Amplified Fragment Length Polymorphisms (AFLPs), SSLPs can provide a fully informative PCR assay. Although Single Nucleotide Polymorphisms (SNPs) have the potential for a significantly greater density, they are generally obtained by comparing two genomic sequences from different strains of the same species, thus requiring significantly more sequencing and assembly to permit identification. Second, candidate SSLPs can be identified from genomic sequence using a bioinformatics-based strategy, dramatically improving the ease and cost-effectiveness; moreover, as sequence assembly progresses, the search for SSLPs can be targeted to sparsely covered regions to improve uniformity of coverage. SSLPs have been used as the basis for genetic linkage maps in many vertebrate species.

Although historically SSLPs have been identified via cloning from small-insert genomic DNA libraries, we have used bioinformatics to identify SSRs within the *X. tropicalis* genome. These SSRs were then tested for polymorphisms between the Nigerian (N) and Ivory Coast (IC) strains of *X. tropicalis*. The resulting SSLPs were then genotyped on a Nc × IC F2 mapping panel to generate a 2886-marker genetic map. Preliminary versions of this map and a database of the SSLP markers have been made available through our website (http://tropmap.biology.uh.edu).

## Materials and methods

### Map cross and F2 DNA panel

The map cross was derived from a single mating of two F1 progeny produced by a P1 cross of a male Nigerian (N) inbred over 7 generations (N7) × a female Ivory Coast (strain IC; inbred from original Adiopoudome collection) inbred over 5 generations (IC5); this initial P1 cross was carried out in the laboratory of Robert Grainger at the University of Virginia. The IC animals represent the same strain as that used in Khokha et al. (2009). The F2 progeny were euthanized as postmetamorphic tadpoles in preparation for DNA isolation. The DNA isolation protocol was modified from Sparrow et al. (2000). Briefly, metamorphic tadpoles were euthanized in 0.05% benzocaine, frozen individually in an ethanol/dry ice bath, and stored at − 80 °C. Each individual was macerated extensively, and the tissues were digested in 100 μg/ml Proteinase K at 55° overnight. Following heat-inactivation of Proteinase K, samples were treated with 50 μg/ml RNase A at 37° for 90 min. DNA was isolated after phenol–chloroform extraction and ethanol precipitation. The DNA isolation protocol can be found at http://tropmap.biology.uh.edu/DNAisolation.html. At least 300 μg of DNA was isolated from each individual. A DNA panel representing 190 F2 individuals as well as the 2 P1 individuals was used to map the SSLPs.

### Bioinformatic identification of unique SSRs

Simple Sequence Repeats (SSRs) were identified within scaffolds from the JGI *X. tropicalis* genome assembly 2.0. Assembly 2.0 was the current assembly at the time this project was initiated, and it consists of over 27,000 scaffolds. SSRs were identified bioinformatically using an algorithm modified from Tandem Repeats Finder (Benson, 1999), which selected a single di- tri-, or tetranucleotide SSR with unique flanking sequences (“unique SSR”) from each scaffold.

### Assessment of polymorphisms

Primers were designed via Primer 3 (Rozen and Skaletsky, 2000; http://www-genome.wi.mit.edu/cgi-bin/primer/primer3_www.cgi) to amplify the SSR and flanking sequence to generate an amplified fragment between 80 and 500 bases under standard conditions. The amplified fragment was then tested via BLAST (McGinnis and Madden, 2004; http://www.ncbi.nlm.nih.gov/BLAST) to determine whether it represented a unique sequence within the genome. All primers were obtained from Illumina (San Diego, CA). Primer sets were tested initially for robust amplification of a single fragment visualized by agarose gel electrophoresis, using the following standard conditions (30 cycles, 58 °C annealing temperature, and 1.5 mM MgCl_2_). SSR primer sets showing robust amplification were then tested for polymorphisms using DNAs from unrelated Nigerian and Ivory Coast individuals as well as a pool of F2 DNAs represented in the map cross. Polymorphism PCR assay reactions included ^32^P-dCTP, and radiolabeled PCR products were visualized following polyacrylamide gel electrophoresis. Sequence length polymorphisms were detectable as differences in the size of the PCR product; these assays also provided an estimate of allelic size differences.

### Genotyping and linkage analysis

Genotyping of identified SSLPs against the F2 mapping panel was carried out via PCR reactions run in duplex, using primers labeled with the fluorochromes FAM and HEX (Illumina, San Diego, CA). PCR products were separated on an ABI 3730 DNA Sequencer. Initial genotyping data were examined using GeneMapper 3.7 software (Life Technologies, Carlsbad CA). Linkage analysis was carried out using JoinMap 3.0 (Van Ooijen and Vorrips, 2001), which can incorporate a wide range of polymorphism types; thus, genotyping results were classified according to categories of polymorphism, as specified by JoinMap 3.0 protocols. Genotyping results were evaluated individually; markers that could not be definitively genotyped were eliminated from the analysis. For comparative purposes, a second linkage analysis was performed using MSTmap (Wu et al., 2008).

### Preparation of metaphase spreads and Fluorescence *In situ* Hybridization (FISH)

Metaphase spreads were prepared from primary cell cultures derived from testes of juvenile frogs (Ivory Coast strain). Mitotic arrest was induced by treatment with 0.2 μg/ml colchicine (Sigma) for 4.5 h at 28 °C in 7% CO_2_ atmosphere. Cells were collected by trypsinization and centrifugation at 100 × *g* for 10 min at room temperature (RT). Hypotonic shock, fixation and spreading of cells were described previously (Krylov et al., 2007). Slides were dried and treated with 50 μg/ml pepsin in 0.01 N HCl for 5 min at 37 °C, followed by a 30-minute incubation in 2% paraformaldehyde at RT. Endogenous peroxidase was quenched by a 30-minutes incubation in 1% hydrogen peroxide. Methods for FISH-TSA, including preparation and labeling of cDNA probes, have been described previously (Krylov et al., 2007).

### Assignment of linkage groups to *X. tropicalis* chromosomes

Assignment of linkage groups (LG) to *X. tropicalis* chromosomes was based on physical mapping of cDNA probes by means of fluorescence *in situ* hybridization coupled with a tyramide amplification step (FISH-TSA) (Khokha et al., 2009; Krylov et al., 2007). Appropriate genes or cDNA clones that were a minimum of 900 bp in length were selected from scaffolds anchored in respective linkage groups using Ensembl (http://www.ensembl.org/indeX.html) or GenBank (http://www.ncbi.nlm.nih.gov/Genbank/) databases. On one occasion (LG6, scaffold 104), the Gurdon Institute Xt. EST database (http://genomics.nimr.mrc.ac.uk/cgi-bin/public.exe) was used as a data source (see Supplementary Table 2). Chromosome identification and numbering were based on the p/q ratio following the revised nomenclature system described in Khokha et al. (2009). The position of the FISH-TSA signal was expressed as a relative distance from the centromere (RDC) with respect to the appropriate arm. Both the p/q arm ratio and RDC were determined using ACC Program v. 5.0 (SOFO, Brno, Czech Republic). At least six mitoses showing a FISH-TSA signal were evaluated for each physically mapped locus.

## Results

### Identification of SSLPs

Over 14,000 SSRs were identified using our data mining script. Several rounds of script modification were required to ensure that the selected markers represented unique sequences. Initially, the script selected only tri- and tetranucleotide repeats; once those were exhausted, the script was modified to include dinucleotide repeats, which are considerably more abundant (Xu et al., 2008).

Over 12,000 SSR primer sets were tested for amplification; of these, 9369 (78%) were robustly amplifiable under our standard conditions. The first 6400 primer sets included primarily tri- and tetranucleotide SSRs. At least one unique tri- or tetranucleotide SSR was identified from all scaffolds containing these sites. Scaffolds containing one or more unique tri- or tetranucleotide SSRs comprise approximately 85% of the genome. The remaining scaffolds were thus not represented within this initial set. The remaining SSRs contain dinucleotide repeats; these are considerably more abundant and amplify more reliably than the tri- and tetranucleotide SSRs.

**Fig. 1:**
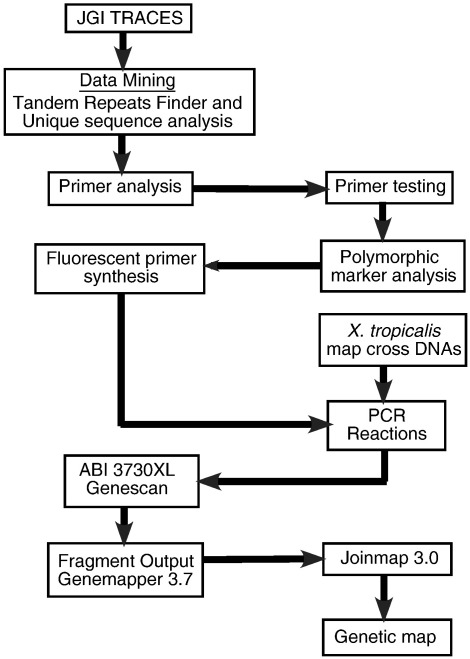
Organization of Bioinformatics, SSLP testing, and genotyping. Data mining of sequence traces from JGI genome assembly 2.0 identified SSRs, which were then tested for PCR amplification and polymorphisms. SSLPs shown to be polymorphic in the initial P1 cross were used to genotype the map cross panel using an ABI 3730 XL sequencer. Genotyping results were scored using Genemapper 3.7, and linkage analysis was carried out using JoinMap 3.0.

Unique SSR sequences were amplified from genomic DNA isolated from the Nigerian and Ivory Coast grandparents (P1 individuals). In some instances, DNA from unrelated Nigerian and Ivory Coast individuals were also included, or DNA from pooled F2 individuals was used instead. Out of 9300 SSRs tested, 5829 (63%) showed polymorphisms between the Nigerian and Ivory Coast individuals, and 4779 SSRs (82% of polymorphic SSRs) were polymorphic between the P1 individuals. A marker database, which includes primer sequences, sequence of the amplified fragment, SSR type, and scaffold assignment, is available at http://www.tropmap.uh.edu. A diagram of the workflow for identification of markers and generation of the genetic map is shown in Fig. 1.

### The F2 map cross panel

A mapping cross F2 DNA panel representing 190 sibling individuals was generated from a single cross of two F1 progeny produced by the P1 cross of a male Nigerian (N) F7 with a female Ivory Coast (IC) F5. A total of 3982 SSRs (83% of the P1 polymorphic set) were genotyped using this DNA panel and DNA samples from each parent; these SSRs had been found to be polymorphic in either the P1 individuals or a pool of the F2 individuals. We were unable to place 1003 (25%) of the genotyped markers on the map; most of the unplaced markers were either difficult to score, did not show polymorphisms in the mapping cross, or were eliminated in preliminary iterations of the map because of anomalies in genotyping or linkage analysis. Of the 2979 polymorphic markers that could be clearly scored, only 93 (3%) were not resolvable on the map; presumably, these markers were too far away (> 20 cM) from other clearly scorable markers to show linkage. The mapping cross represents 380 informative meioses, and thus the resulting map has a theoretical limit of resolution of 0.26 cM.

### Genotyping analysis

Primary linkage analysis was carried out using JoinMap 3.0 (Van Ooijen and Vorrips, 2001). JoinMap uses maximum likelihood to calculate recombination frequencies between all possible pairs of markers; from this data set, it assigns linkage groups, predicts marker order within linkage groups, and calculates the intermarker distance (Stam, 1993). JoinMap is particularly suitable for our studies because it permits a greater range of heterozygosity in parental genotypes, which allows us to use as many of the SSLPs as possible. It has been used extensively to create genetic linkage maps in plants (e.g., Kuittinen et al., 2004; Song et al., 2004). After an initial analysis that divided the markers into linkage groups, each linkage group was analyzed individually; upon completion of a linkage analysis in Joinmap, the results were compared with earlier versions of the map (i.e., preliminary versions prepared with fewer markers), and the genotyping results for selected markers were reviewed individually. In most cases, several markers were eliminated from a given linkage group, and the remaining markers were re-analyzed. With the exception of LG1, each of the major linkage groups was analyzed more than once.

Linkage analysis yielded a genetic map of 2886 SSLP markers, organized in 10 major and 4 minor linkage groups. The linkage groups included 103–506 markers, in groups ranging from 77 to 138 centimorgans (cM). The LOD scores at which these linkage groups appeared ranged from 3 to 12. The 4 minor linkage groups included a total of 54 markers in groups ranging from 6 markers over 7 cM to 29 markers across 40 cM. A comparison of the linkage groups in terms of size, number of markers, and minimal genomic representation is shown in Table 1. A spreadsheet of the complete genetic map is presented in Supplementary Table 1; this spreadsheet also includes the primers and amplified sequence for each marker, as well as the corresponding scaffold from *X. tropicalis* genome assembly 4.1 (Hellsten et al., 2010).

The total genetic interval across all linkage groups is 1097.64 cM. Since a mutation can be mapped if it is within 20 cM of a marker, the effective size of the map includes not just the total genetic interval, but also an additional 20 cM at either end of each linkage group (Knapik et al., 1996). Thus, the effective size of the map is approximately 1658 cM.

### Assignment of linkage groups to chromosomes

**Fig. 2:**
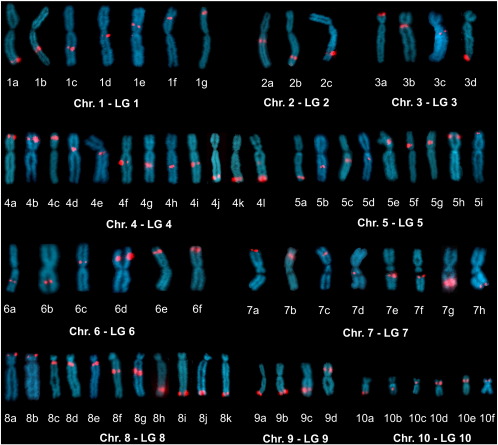
Assignment of linkage groups to *Xenopus tropicalis* chromosomes via FISH-TSA using following cDNA probes. **LG1: 1a.***pias2* (q 0.95), **1b.***cabin1* (q 0.54), **1c.***dao* (q 0.48), **1d.***adh1b* (q 0.22), **1e.***camk2d* (p 0.25), **1f.***exoc1* (p 0.54), **1g.***whsc2* (p 0.78). **LG2: 2a.***trit1* (q 0.28), **2b.***uspl1* (q 0.75), **2c.***pan3* (q 0.76). **LG3: 3a.***jhdm1d* (p 1.00), **3b.***phf15* (q 0.10), **3c.***fgfr4* (q 0.26), **3d.***chd3* – cluster F (q 1.00). **LG4: 4a.***sox6* (p 0.96), **4b.***ext2* (p 0.83), **4c.***acp2* (p 0.68), **4d.***rasgrp2* (p 0.23), **4e.***adamts18* (q 0.14), **4f.***e2f4* (q 0.32), **4g.***mast2* (q 0.45), **4h.***pcsk9* (q 0.47), **4i.***dmap1* (q 0.48), **4j.***rybp* (q 0.87), **4k.***ppp4r2* (q 0.90), **4l.***ptprg* (q 0.96). **LG5: 5a.***tram2* – cluster A (q 0.88), **5b.***ap2m1* (q 0.47), **5c.***myo6* (q 0.35), **5d.***cdc40* (q 0.25), **5e.***crim1* (p 0.26), **5f.***ttc27* (p 0.30), **5g.***cyp1b1* (p 0.41), **5h.***prepl* (p 0.77), **5i.***bre* (p 0.93). **LG6: 6a.***atp6v1h* (q 0.55), **6b.***b4galt6* (q 0.54), **6c.***fignl1* (p 0.24), **6d.** Xt7.1-THdA017i08.3 (p 0.65), **6e.***mpp7* (p 0.68), **6f.***csrnp1* (p 0.85). **LG7: 7a.***nop2* (p 0.92), **7b.***gpr123* (p 0.59), **7c.***got1* (p 0.46), **7d.***mfn2* (q 0.14), **7e.***cbl* (q 0.43), **7f.***tbcel* (q 0.43), **7g.***fut1* (q 0.78), **7h.***agmat* (q 0.95). **LG8: 8a.***glipr2* – cluster 5B (p 0.92), **8b.** ENSXETG00000000667 (p 0.83), **8c.***dusp9* (q 0.04), **8d.***smc1a* (q 0.04), **8e.***nr6a1* (q 0.09), **8f.***rps6ka6* (q 0.36), **8g.***f9* (q 0.40), **8h.***zfp36l1* (q 0.78), **8i.***mef2d* (q 0.90), **8j.***rhbg* (q 0.92), **8k.***ubqln4* (q 0.93). **LG9: 9a.***smarcal1* (q 1.00), **9b.***znf142* (q 0.98), **9c.***tbr1* (q 0.74), **9d.** MGC145260 (p 0.58). **LG10: 10a.***map2k4* (q 1.00), **10b.***src* (q 0.68), **10c.***nmt1* (p 0.28), **10d.***hoxb3* (p 0.51), **10e.***sp2* (p 0.53), **10f.***taf4* (p 0.75).

Fluorescence *in situ* hybridization coupled with a tyramide amplification step (FISH-TSA) was used for the physical mapping of cDNA probes in order to assign linkage groups to *X. tropicalis* chromosomes. In total, 69 cDNA markers were localized (Fig. 2). A detailed description of mapped loci is provided in Supplementary Table 2. Several DNA probes were used for each LG, and probes for a single LG mapped to only one chromosome. A gynogynetic mapping strategy has previously been used to identify the locations of the centromeres and markers corresponding to centromeric positions (Khokha et al., 2009). LGs 2, 3, 4, 7, and 8 showed a p–q orientation, while the remaining linkage groups were oriented from q to p. All of the minor linkage groups were assigned to chromosomes; they are listed as LG3b, 5b, 8b, and 8c. Assignments of 3b, 5b, and 8b were based on the position of probes representing scaffolds included solely within those minor linkage groups; assignment of 8c is based on the position of a marker from Scaffold 427, which is represented both in LG8 and in 8c.

**Fig. 3:**
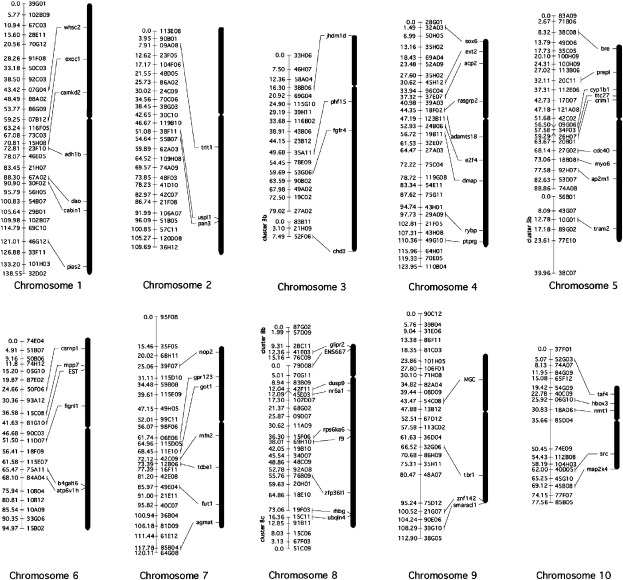
Alignment of Physical and Genetic maps. Each of the ten chromosomes of *X. tropicalis* is depicted proportional to its size and centromere location. Locations of genes on each chromosome are shown based on *in situ* hybridization results. Representative markers and their centiMorgan positions from each of the linkage groups are shown alongside the chromosomes. The *in situ* mapped chromosomal genes are aligned to their closest linkage group marker. This linkage was established by comparing the location of the genetic markers and the location of the mapped genes within the same genomic scaffold.

With the exception of LG2, all linkage groups covered the length of the corresponding chromosomes (Fig. 3). Cytogenetic mapping of LG2 showed that this linkage group covers the q arm of Chr. 2, terminating near the centromere. The p arm of Chr. 2 is thus not represented in this genetic map.

Several scaffolds have been cytogenetically mapped to the p arm of Chr. 2; these will be described elsewhere (Mácha et al., manuscript in preparation). Markers representing these scaffolds were identified from our marker dataset, and the genotyping results for these markers were assessed. Of the genotyped markers from these scaffolds, 73% were nonpolymorphic in our F2 mapping panel. To establish a size-matched “control” dataset, we also identified markers from the scaffolds that flanked each of the Chr.2 p arm scaffolds in size, i.e., if scaffold 40 was represented on the Chr. 2 p arm, scaffolds 39 and 41 were included in the control dataset. We then reviewed the genotyping results for all markers from scaffolds in the control dataset; only 11% of these markers were nonpolymorphic across the F2 mapping panel, which is significantly different (p < 0.0001, Fisher's Exact Test) from that found for the markers representing the Chr.2 p arm scaffolds. These findings suggest that the level of polymorphism on the p arm of Chr. 2 is substantially reduced relative to the regions represented on the map.

### The genetic map and the genome assembly

We investigated the correspondence between genetic interval and genomic sequence by comparing the maximum genetic interval and genomic distance between markers on 74 of the largest 100 scaffolds; the remaining 26 scaffolds in this group were either represented by a single marker or were otherwise unsuitable. The ratio of genomic distance to genetic interval varied over 3 orders of magnitude, from approximately 4 kb/cM to over 3000 kb/cM; the average value is 581 kb/cM, comparable to the value of 625 kb/cM for zebrafish (Postlethwait et al., 1994). Interestingly, this value was not strongly correlated with distance from the centromere; scaffolds in proximity to the centromere included those with low ratios, as well as the expected higher kb/cM values, and some of the scaffolds with values over 1000 kb/cM were found far from the centromeric region (data not shown). Moreover, one-way ANOVA indicated that there were no significant differences in the average map interval/scaffold ratios for all scaffolds among the individual linkage groups (data not shown). It should be noted, however, that the order of markers from a single scaffold on the genetic map often did not correspond to marker order in the genomic sequence within a scaffold, and that markers from a single scaffold were generally intermixed with markers from other scaffolds in the genetic map. Thus, for close-range analysis, the overall correspondence of marker order between the genetic map and the genomic sequence is relatively weak.

The striking short-range intermixing of markers from different scaffolds led us to ask whether this was a general feature of the map, or whether some linkage groups had a higher level of disjunct (i.e., intermixed) markers than others. As an indicator of “intermixing”, we assessed each scaffold represented by more than one marker and identified the numbers of disjunct vs conjunct scaffolds for all scaffolds represented by only two markers (data not shown). The number of 2-marker scaffolds in each major linkage group (LG1–10) ranged from 5 to 27. For the 7 linkage groups with at least 10 2-marker scaffolds, the frequency of conjunct 2-marker scaffolds (i.e., scaffolds in which the representative markers are placed sequentially on the map, without intervening markers from other scaffolds) ranged from 26 to 36%; overall, 30% of 2-marker scaffolds were conjunct. We also compared the genetic distance between the representative markers for all of the 2-marker scaffolds for each linkage group (data not shown). For individual linkage groups with at least 10 2-marker scaffolds, between 50 and 72% were represented across a distance of 1 cM or less. Across all of the major linkage groups, over 60% of the 2-marker scaffolds are represented within a genetic distance of 1 cM. We tentatively conclude that the frequency of scaffold intermixing is relatively constant across the map and thus cannot be attributed to the specific properties of any individual linkage group.

### Genomic representation

We estimated the minimum genomic representation for each linkage group by identifying the scaffolds from Assembly 4.1 that contained each of the mapped markers and determining the total size of scaffolds represented unambiguously in each linkage group. The minimum genomic representation varied between 29 and 164 MB across the 10 linkage groups.

Each linkage group included markers for which no single matching scaffold from Assembly 4.1 could be identified. These included markers that corresponded to sequences in multiple scaffolds, as well as markers that did not match any of the scaffolds from Assembly 4.1. Since each of the SSLP markers originated as a unique sequence from Assembly 2.0, these sequences have presumably been lost from the later genome assembly. The entire genetic map includes 126 sequences (4.3% of markers) with multiple corresponding scaffolds, and 122 sequences (4.2%) with no corresponding scaffold.

Scaffold matches for the remaining markers allowed us to assign individual scaffolds to linkage groups. For a scaffold to be assigned to a linkage group, 75% of markers included on the scaffold had to map to a single linkage group. The 45 scaffolds that did not meet this criteria were left “unassigned”, as listed in Table 1; they constitute just over 87 MB of the total genomic representation on the genetic map. The minimal genomic representation of the entire map totaled 1.129 GB, or 66% of the 1.7 GB represented in *X. tropicalis* genome assembly 4.1; the size of the assembled genome is similar to the genome sizes corresponding to the c-values obtained for *X. tropicalis* (1.47–1.88 GB; Gregory, 2006). Approximately half of the *X. tropicalis* genome assembly is represented in the first 272 scaffolds (Hellsten et al., 2010); all but 38 of these scaffolds are represented in the genetic map.

Scaffolds in the unassigned group included markers that mapped to multiple linkage groups. To evaluate the provenance of these scaffolds, we checked the associated marker sequences for their scaffold assignments in *X. tropicalis* genome assembly 5, a test genome assembly that was assembled using the ARACHNE compiler at the Stanford Genome Center. Out of the 45 “unassigned” Assembly 4.1 scaffolds, 30 included markers that were incorporated into different scaffolds in Assembly 5, suggesting that these 30 scaffolds from Assembly 4.1 may be chimeric. For 8 of the unassigned scaffolds, one or more SSLPs within the marker set could not be assigned to a single Assembly 5 scaffold, so they could not be evaluated. The remaining 7 scaffolds were cohesive, meaning that all markers within a given Assembly 4 scaffold mapped to one Assembly 5 scaffold. This result indicates that for these 7 cohesive scaffolds, there is a discrepancy between the genome assembly, which demonstrates that these sequences are in physical proximity to one another, and the genetic map, which places markers from a single scaffold into different linkage groups. We do not have an explanation for these findings, but caution those using this map for positional cloning studies to avoid using these scaffolds as a major source of linkage relationships. A list of unassigned scaffolds and their status with regard to assembly 5 is provided in Supplementary Table 3.

### Alternative linkage analysis using MSTmap

In view of the discrepancies between the genetic map and genome assembly 4.1, we carried out a second linkage analysis using MSTmap, an alternative mapping program that establishes marker order using an algorithm based on the “minimal spanning tree” (MST) of a graph of the genotyping data (Wu et al., 2008). This alternative analysis required a significant recoding of the genotype data. In JoinMap 3.0, the F2 alleles were grouped in 5 classes based on the P1 genotype; each P1 genotype could generate 2–4 different F2 allelic combinations, depending on the number of alleles shared between the parents, for a total of 14 different types of F2 allelic combinations. In contrast, MSTmap recognizes only 3 types of F2 allelic combinations; thus, the transformation of these data into MSTmap format significantly reduced the complexity of the data set.

MSTmap analysis of the recoded genotypes yielded 96 linkage groups that included between 3 and 261 markers, 1 pair of linked markers, and 1 unlinked marker, incorporating 2326 markers in all. The total size of the map, representing the sum of the sizes of all linkage groups, is 1991.8 cM. Each MSTmap linkage group (lg) consisted of markers that could be found on a single linkage group from the primary Joinmap analysis (referred to as “LGs”); however, the markers within a single MSTmap lg cover large distances on the original Joinmap LGs, and the MSTmap lgs show considerable overlap on the corresponding Joinmap LGs. Interestingly, the MSTmap lgs tended to represent groupings of markers that displayed the same type of allelic combination in the original genotype data, suggesting that the MSTmap algorithm “sorts” markers by allelic combination as a byproduct of the computational process, despite the fact that specific information regarding the allelic combination for a given marker is lost during recoding. A spot-check comparison of the correspondence between the genome assembly and either of the two genetic maps for 38 scaffolds indicated that for 5 scaffolds, the markers from a given scaffold showed improved marker order (markers are more conjunct, i.e., less intermixing with markers from other scaffolds) in the MSTmap lgs. For 20 scaffolds, the markers were more conjunct in the Joinmap LGs, and for 13 scaffolds, the number of conjunct markers was identical in both linkage analyses (data not shown). Thus, in our view, while the MSTmap lgs confirm linkages shown by Joinmap, the MSTmap analysis does not produce the hoped-for improvement in marker order. We include the MSTmap linkage groups as a supplement (see Supplementary Table 4), as they may provide some alternative candidate linked markers.

We have not been able to combine MSTmap lgs that are included within the same Joinmap LG into a single linkage group in MSTmap. It is likely that the high level of parental heterozygosity for most of these markers precludes the detection of linkage using the MSTmap algorithm.

## Discussion

We have generated an SSLP map of over 2800 markers, with an effective size of 1658 cM and a theoretical limit of resolution of 0.26 cM. This map is the third version that has been released through our website (http://tropmap.biology.uh.edu). A comparison of the size of the current 2886-marker map with the previous 1654-marker map suggests that 1658 cM may be near the maximum size of the genetic map, since the effective size of the previous map was 1636 cM. Thus, a 74% increase in the number of markers has yielded an increase of less than 2% in map size.

The minimal genomic representation of the entire map totaled to 1.129 GB, or 66% of the 1.7 GB represented in *X. tropicalis* genome assembly 4.1; the size of the assembled genome is similar to the genome sizes corresponding to the c-values obtained for *X. tropicalis* (1.47–1.88 GB; Gregory, 2006).

This map demonstrates that a bioinformatics-based strategy is an effective means of identifying SSLPs. The frequency of unique, usable SSLPs identified in our study was high relative to those of SSLPs identified via conventional CA-repeat screening of genomic libraries. Our recent analysis of 25% of the SSRs in the *X. tropicalis genome* (Xu et al., 2008) has shown that the frequency of all di-tri and tetranucleotide SSRs in *X. tropicalis* is close to 1/6 kb, much higher than previously suggested. Moreover, a bioinformatics strategy can be targeted to specific scaffolds to expand coverage of the genome.

A provisional AFLP map for *X. tropicalis* has been published (Kochan et al., 2003). This map includes 51 AFLP markers and 2 isozyme markers in 13 linkage groups. Since no sequence information is available for the AFLP markers, it is not possible to integrate this map with either our map or the genome assembly. Future mapping efforts may focus on SNP identification and mapping; since our strategy provides a correspondence between the genetic map with the genome sequence, it should be possible to integrate our map with future SNP mapping results.

This map shows two major discrepancies with the physical map. First, 8.5% of the markers on this map cannot be uniquely identified on a single scaffold from the current assembly (4.1; Hellsten et al., 2010); some markers are not represented on any scaffold in this assembly, while others show close matches to multiple scaffolds. Most of these are resolved in *X. tropicalis* genome assembly v6; comparisons to v6 indicate that only 38 markers cannot be unambiguously assigned to a single scaffold (Gilchrist and Zimmerman, pers. comm.). Second, there are discrepancies in short-range marker order, in that markers assigned to a single scaffold are intermixed along the genetic map with markers from other scaffolds; in many cases, these represent intermixing over short genetic distances. Most of these disjunct scaffolds are not resolved in later assemblies (Gilchrist and Zimmerman, pers. comm.). During our linkage analyses, we found that, although long-range marker position was robust, short-range marker position was often exquisitely sensitive to the presence of individual markers in flanking regions. Comparisons of genetic and 2 different sequence-based physical maps of the human genome suggested that most of the discrepancies could be attributed to errors in genome assembly (DeWan et al., 2002); however, this study was restricted to a limited set of markers that passed a more stringent test of marker position (likelihood ratio). The spatial heterogeneity of recombination frequencies along each chromosome may contribute to the apparent intermixing of markers within the genetic map. Increases in marker density on the genetic map should improve the short-range colinearity of these resources. Colinearity between the map and genome has already been increased by improvements to the genome assembly: the newly-released Assembly 7.1 shows greatly improved colinearity with the genetic map (Schmutz, Jenkins, and Rokhsar, personal communication). It should be noted, however, that the genetic map was incorporated in the construction of Assembly 7.1.

Graf (1989) generated a preliminary set of 8 linkage groups for *X. laevis* using 21 isozyme markers. We have attempted to compare these linkage groups to our map by finding the scaffold and linkage group assignments for the corresponding *X. tropicalis* genes. In many cases, the corresponding genes could not be unambiguously identified. We were, however, able to determine that the linkage relationships represented in Graf's linkage groups 1 and 6 are not conserved, although the 3 isozyme markers that constitute Graf's linkage group 7 are all found within our LG2 (data not shown). Further conclusions regarding the conservation of organization between the *X. tropicalis* and *X. laevis* genomes await sequencing and assembly of the *X. laevis* genome.

The striking absence of coverage on the p arm of Chr. 2 indicates that we have been unable to detect linkage either to or within this area. Although this omission could occur for a number of reasons, our results suggest that the level of polymorphism is lower in this region than in the regions of the genome that are represented on the genetic map. Cytogenetic analyses of *X. tropicalis* lampbrush chromosomes (Penrad-Mobayed et al., 2009) should reveal any large-scale differences in crossover frequency for the p arm of Chr. 2. One explanation for a reduced frequency of polymorphism is “selective sweep”, in which recent strong selection for an allele increases the frequency of alleles in regions flanking the locus at which selection occurs, via “genetic hitchhiking” (Dorit et al., 1995). Selective sweep is thought to underlie a dramatic reduction in polymorphisms over a 1-MB region in the maize genome, in comparison with its undomesticated relative teosinte, another subspecies of Zea mays (Tian et al., 2009). Analysis of single nucleotide polymorphism (SNP) distribution in the human genome has identified several genomic regions in which reduction of polymorphism may represent a selective sweep, including 2 areas located on the X chromosome (International HapMap Consortium, 2005). Studies of the Segregation Distorter (SD) complex in African populations of Drosophila melanogaster have demonstrated a recent selective sweep within the SD-mal variant, which carries a double inversion incorporating the region of the SD complex; on the SD-mal chromosome, recombination is suppressed across 14 MB (Presgraves et al., 2009). These authors point out that selfish meiotic drive complexes such as SD could become fixed in the absence of modifying loci or counteracting selection, leading to regional suppression of recombination. Although selective sweep alone is unlikely to explain the omission of the entire p arm from the genetic map, a localized region subject to selective sweep might disrupt our ability to detect linkage relationships over a larger area. Moreover, this relationship suggests that the p arm of chromosome 2 may harbor genes or gene complexes that could underlie previously undetected meiotic drive.

Suppression of recombination may occur in regions associated with sex determination. Although frogs use a ZW sex determination system, the Z and W chromosomes are morphologically indistinguishable (for review see Evans, 2008). A recent study indicates that 3 genes known to be sex-linked in the anuran Rana rugosa are located on *X. tropicalis* Chr. 8 (Tymowska Chr. 10) (Uno et al., 2008). The DM-W gene is essential for ovary development in *X. laevis* and may function in sex determination; bioinformatic comparisons, however, indicate that DM-W cannot be identified in the *X. tropicalis* genome (Yoshimoto et al., 2008). The locus responsible for sex determination in *X. tropicalis* remains unknown. Identification and sequencing of sex-linked AFLPs in *X. tropicalis* have been used to identify 9 scaffolds that show sex linkage (Olmstead et al., 2010); 4 of these are represented in LG7, and the remainder is not represented on the map.

This map will be a significant resource for genetic and genomic studies of *X. tropicalis*. Preliminary versions of this map have already been used to map genes identified by mutation (Abu-Daya et al., 2009) and track the movement of transposable elements (Yergeau et al., 2010). The genetic map, together with the marker-scaffold correspondence, has also contributed to the evaluation of predicted syntenic relationships and long-range assembly of the *X. tropicalis* genome (Hellsten et al., 2010). Given the map size, resolution, marker density, and relative ease of use, the map should be valuable for the integration of genetics with genomic or cytogenetic studies.

The following are the supplementary materials related to this article.

Supplementary Table 1 The *X. tropicalis* genetic map. This table provides the linkage groups, map positions, Assembly 4.1 scaffold assignments, PCR primers, sequences, and amplified fragment sizes for each marker on the map. In addition, the full marker ID is included, which can be used to search the map database for additional information (http://tropmap.biology.uh.edu).

Supplementary Table 2 Probes used for FISH-TSA. The table lists the probes used for cytogenetic mapping of each linkage group. It includes the Assembly 4.1 scaffold assignment, gene, PCR primers, and position on the chromosome for each probe. RCD, relative distance from centromere.

Supplementary Table 3 Unassigned scaffolds. This table lists the scaffolds that are represented by markers on multiple linkage groups, with fewer than 75% of the markers on a single linkage group. For provenance in Assembly 5, “multiple” indicates that markers from a single Assembly 4 scaffold are segregated into multiple scaffolds in Assembly 5; “single” indicates that all markers from the assembly 4 scaffold are found on a single Assembly 5 scaffold; “undetermined” indicates that not all markers from the assembly 4 scaffold could be identified in Assembly 5.

Supplementary Table 4 Linkage groups generated in MSTmap. The table shown on the first sheet provides a summary of the linkage groups obtained by analysis using MSTmap, including the number of markers and size for each linkage group, as well as the corresponding JoinMap linkage group in which the MSTmap linkage group is found. The table on the second sheet lists each MSTmap linkage group consecutively, including the markers and map positions.

## Figures and Tables

**Table 1:** Linkage Groups in 2886-Marker Map.

LG	Chr.^a^	Old LG^b^	Total cM^c^	# markers	#scaffolds^d^	Genomic representation^e^(kB)	Avg intermarker dist (cM)
1	1	1	138.55	506	141	164,091.4	0.27
2	2	6	109.69	287	79	96,509.8	0.38
3	3	8	79.02	264	70	93,694.2	0.30
4	4	7	123.95	365	90	121,131.2	0.34
5	5	9	88.86	207	56	67,565.4	0.43
6	6	2	94.97	357	87	120,335.4	0.27
7	7	4	120.11	303	99	92,423.4	0.40
8	8	5	73.06	264	77	91,586.8	0.28
9	9	3	112.9	178	50	68,777.6	0.64
10	10	10	77.56	99	30	28,636.7	0.75
5b	5	A	39.96	29	15	81,869.5	
8c	8	E	16.36	12	8	3,978.0	
3b	3	F	7.49	8	5	5,881.6	
8b	8	5b	15.16	7	4	4,407.7	
Add′l^f^	–		–		45	87,379.0	
Total			1097.64	2886	856	1,128,267.7	

a Chromosomes are numbered as described in Khokha et al. (2009).

b “Old LG” refers to the initial linkage group designation previously shown on our website. All linkage groups are now numbered in accordance with the corresponding chromosome.

c “Total cM” refers to the distance from the first to the last marker within a linkage group.

d Only those scaffolds that are represented entirely within a single linkage group are included in the “# of scaffolds represented” for each linkage group.

e “Genomic representation” refers to the combined size of all scaffolds represented within a linkage group; this number is a minimum estimate of the portion of the genome represented within a linkage group.

f The total minimal genomic representation includes approximately 87 MB from scaffolds that appear on 2 or more linkage groups (“Add′l”).
