# Supplementary material for: A genetic map of Xenopus tropicalis
Source: Dev Biol. 2011 Jun 1;354(1-2):1–8. doi: 10.1016/j.ydbio.2011.03.022 (PMC3098391; doi:10.1016/j.ydbio.2011.03.022)
Supplement: Supplementary Table 3 — Unassigned scaffolds. This table lists the scaffolds that are represented by markers on multiple linkage groups, with fewer than 75% of the markers on a single linkage group. For provenance in Assembly 5, “multiple” indicates that markers from a single Assembly 4 scaffold are segregated into multiple scaffolds in Assembly 5; “single” indicates that all markers from the assembly 4 scaffold are found on a single Assembly 5 scaffold; “undetermined” indicates that not all markers from the assembly 4 scaffold could be identified in Assembly 5. [file mmc3.doc]

**Supplementary Table 3. Unassigned Scaffolds.**

| **scaffold** | **Represented in LGs** | **Assembly 5 scaffolds** |
| --- | --- | --- |
| 2 | 5, 9 | multiple |
| 8 | 1, 5 | single |
| 21 | 6, 9 | multiple |
| 34 | 3, 6 | multiple |
| 43 | 1, 10 | multiple |
| 59 | 1, 6 | multiple |
| 75 | 1, 7 | multiple |
| 76 | 1, 5 | multiple |
| 106 | 7, 2 | multiple |
| 110 | 1, 7 | multiple |
| 112 | 1, 4 | multiple |
| 144 | 3, 5b, 6, 10 | multiple |
| 165 | 7, 10 | undetermined |
| 167 | 1, 2, 6 | multiple |
| 197 | 3, 6 | multiple |
| 209 | 2, 7 | single |
| 211 | 3, 4, 6 | multiple |
| 241 | 2, 8 | multiple |
| 252 | 4, 5 | multiple |
| 253 | 2, 3 | multiple |
| 261 | 2, 4 | multiple |
| 263 | 6, 7 | single |
| 270 | 4, 7 | multiple |
| 273 | 3, 8, 9 | multiple |
| 279 | 4, 8c | undetermined |
| 332 | 8, 10 | multiple |
| 357 | 2, 9 | multiple |
| 377 | 2, 6 | multiple |
| 393 | 1, 7 | undetermined |
| 428 | 5b, 7 | multiple |
| 468 | 1, 7 | single |
| 505 | 1, 2 | multiple |
| 523 | 1, 2 | multiple |
| 561 | 5, 6 | single |
| 569 | 1, 5 | undetermined |
| 601 | 1, 3 | multiple |
| 614 | 5, 9 | single |
| 617 | 1, 4, 8 | multiple |
| 644 | 1, 7 | multiple |
| 650 | 2, 7 | multiple |
| 717 | 1, 9 | undetermined |
| 739 | 3, 5b | undetermined |
| 830 | 4, 10 | undetermined |
| 863 | 1 ,4 | single |
| 1423 | 4, 7 | undetermined |
